# MANAGING MANAGED CARE: PERSPECTIVES FROM KEY STAKEHOLDERS IN SKILLED NURSING FACILITIES

**DOI:** 10.1093/geroni/igz038.1846

**Published:** 2019-11-08

**Authors:** Andrea E Daddato, Cynthia Drake, Edward A Miller, Pamela Nadash, Denise Tyler, Rebecca S Boxer

**Affiliations:** 1 University of Colorado School of Medicine, Aurora, Colorado, United States; 2 Colorado School of Public Health, Aurora, Colorado, United States; 3 University of Massachusetts Boston, Boston, Massachusetts, United States; 4 RTI International, Waltham, Massachusetts, United States; 5 Kaiser Permanente Institute for Health Research, Aurora, Colorado, United States

## Abstract

In recent years, Medicare Advantage (MA) plan enrollment has increased, a trend that is expected to continue. Many skilled nursing facilities (SNFs) rely on MA managed care insurer referrals to maintain their census in a market with high competition for post-acute care patients. This study used semi-structured interviews to describe the relationship between MA plans and SNFs from the perspective of key decision-makers in SNFs. Twenty-three interviews were conducted with key stakeholders from 11 Denver Metropolitan area SNFs. A combined purposive-snowball sampling approach was used to identify and recruit select staff from the participating facilities. Interviews focused on the relationship between MA plans and SNFs, including mechanisms of control, power dynamics, and preferences for MA versus Fee-for-Service (FFS) Medicare patients. Key findings included: 1) challenges SNF staff had navigating MA plans’ case management processes, a key mechanism used by MA plans to influence the behavior of SNF decision-makers; 2) MA plans exercising power over beneficiaries’ length of stay, potentially leading to early discharge and heightened risk for rehospitalization; 3) SNF preference for admitting Medicare FFS over MA patients due to higher rates of Medicare FFS reimbursement and greater control over patient care. SNFs are increasingly reliant on MA plans for patient referrals and revenue. The themes suggest that this growing reliance may place SNFs at odds with MA plans on how best to manage overall patient care. It is therefore important that future research investigate how MA plans’ influence over care affects patient outcomes in SNFs and other post-acute settings.

